# Factors Associated with Successful Aging among Community-Dwelling Older Adults Based on Ecological System Model

**DOI:** 10.3390/ijerph17093220

**Published:** 2020-05-06

**Authors:** Hye-Young Jang

**Affiliations:** College of Nursing, Hanyang University, Seoul 04763, Korea; white0108@hanyang.ac.kr; Tel.: +82-2-2220-1781

**Keywords:** successful aging, older adults, ecological system, community

## Abstract

This study was conducted to identify the factors associated with successful aging in older adults based on the ecological system model. Data from the 2017 National Survey of the Living Conditions of Korean Elderly were used. Participants comprised 10,074 older adults. The three principal components in the successful aging model developed by Rowe and Kahn, “absence of disease and disease-related disability,” “maintenance of high mental and physical function,” and “continued engagement with life,” were used to determine successful aging. The collected data were analyzed using descriptive statistics, chi-squared test, t-test, and logistic regression. The study results showed that the correlation factors were age, sex, educational level, economic status, heavy drinking, subjective health status, and health screening in the individual system; living arrangement, satisfaction with spouse, and frequency of contacting family, siblings, and relatives in the family system; and the frequency of contacting neighbors and friends, number of close neighbors and friends, and accessibility of neighborhood facilities in the community system. This study is significant because it confirms that individual characteristics and the environmental systems surrounding older adults should be considered for successful aging; it is necessary to develop and apply healthcare intervention programs that consider both of these aspects.

## 1. Introduction

The older adult population is rapidly growing worldwide. The population of older adults aged 65 or over in the world is 733 million as of 2019 and is expected to double to 1.5 billion by 2050 [1]. Accordingly, a rapid increase in the aging population leads to the perception that a change in perspective on old age is necessary. In other words, there is a need to deviate from the negative perspective of old age associated with illness, weakness, and loss of functions, and focus more on healthy aging and improving the quality of life in old age [2].

Thus, instead of considering older adults as passive dependents who need care due to illness and loss of function, they should be perceived as active individuals who have potential to bring positive changes for spending later years of their life happy and healthy [3]. Hence, the concept of successful aging is becoming more important in adopting a positive perspective on old age [4]. Rather than a single dimensional concept, successful aging is a multidimensional concept, which encompasses physical, social, psychological, and spiritual aspects in addition to culture and environment [5,6]. After being first introduced in 1986 at the annual meeting of the Gerontological Society of America, successful aging has become a core concept in research on aging in which many researchers are engaged [7,8,9]. 

Since the beginning of research on the components of successful aging [3,6], numerous research on the awareness of successful aging [10,11], factors influencing successful aging [12,13,14,15], and the development of a tool for measuring successful aging [16,17,18,19] have been widely conducted in various areas to help understand the concept. 

However, most researchers who systematically analyzed the studies on successful aging made similar conclusions: the concept of successful aging is defined differently by each researcher which causes difficulty in finding consistent predictive factors since there is no set definition of the concept. Thus, further studies are still needed to support the agreed definition of successful aging [6,9,20,21,22] Moreover, most studies acknowledged that successful aging is a multidimensional concept, however, without sufficient argument. Integrated approaches and multidimensional measurements should be sought to enhance successful aging because the factors influencing successful aging are diverse and complex. In particular, experience with successful aging must be taken into consideration as it is affected by the environment in which older adults live [23]. 

The perspective of the ecological system theory takes a multidimensional approach, while focusing on the interaction between humans and the environment, which is useful for understanding successful aging in older adults [24]. The ecological system theory explores different situations in which human behaviors occur and emphasizes the complex interaction between an individual and the environment [24]. In terms of using the ecological system theory, researchers have different opinions on categorizing the concentric structures of the environment that affects individuals and individual development [24,25,26]. Particularly, Zastrow and Kirst-Ashman [26] subdivided the ecological system simply into a microsystem, mesosystem, and macrosystem for clarity. A microsystem includes an individual’s biological, psychological, and social settings, while a mesosystem includes small-scale groups such as family, neighbors, and co-workers that surround the individuals. Lastly, a macrosystem includes community, country, and culture.

Successful aging in older adults should be studied comprehensively from all environmental systems surrounding them, in addition to individual characteristics, in which an individual’s psychological characteristics and the changes in the social environment as well as in the family relationship must be taken into consideration for improving successful aging [17].

Therefore, the purpose of this study was to identify the factors associated with successful aging in community-dwelling older adults based on the ecological system model.

## 2. Methods 

### 2.1. Study Design

This study is a descriptive, secondary analysis research investigating the factors associated with successful aging in community-dwelling older adults based on the ecological system model using the raw data of the 2017 National Survey of the Living Conditions of Korean Elderly (NSLCKE). 

### 2.2. Data and Ethical Considerations

In accordance with the regulations on disclosing and managing raw data of the Korea Institute for Health and Social Affairs (KIHSA), the raw data of the 2017 NSLCKE were provided for this study. The raw data were provided without personal identification information. The NSLCKE is conducted every three years by the national government, in which the collection of raw data was approved by the National Statistics Office (Approval No. 11771) and was performed from 12 June to 28 August 2017. The target population of the 2017 NSLCKE was older adults aged 65 or over who lived in communities in 17 cities and provinces across the country. A total of 10,299 individuals participated in the survey. 

Among the entire 10,299 participants, 10,074 subjects were chosen for the analysis after excluding those who were hospitalized in long-term care facilities (n = 64) and those with missing values in variables related to successful aging (n = 161). 

The NSLCKE was approved by the institutional review board of KIHSA based on Korea Bioethics Law (No. 2017-11). 

### 2.3. Measurements

#### 2.3.1. Criteria for Determining Successful Aging

The three principal components in the successful aging model developed by Rowe and Kahn [27], “absence of disease and disease-related disability,” “maintenance of high mental and physical function,” and “continued engagement with life,” were used to determine successful aging. Participants who met all of the three domains’ criteria fell in the category defined as “successful aging” (Table 1). The single domain of successful aging was operationalized based on previous studies as follows.

(1) Absence of Disease and Disease-Related Disability

For the “absence of disease and disease-related disability” domain, the number of chronic diseases and disabilities were measured. The number of chronic diseases is the number of diseases diagnosed by a doctor. The average number in this study was 2.74 (range: 0–14). The subjects satisfied the criteria if the number of chronic diseases was two or less, based on the average number of chronic diseases in older adults in Korea [28]. The subjects satisfied the criteria if they had not received disability grading. It was determined as successful aging if the two indicators were satisfied in this domain. 

(2) Maintenance of High Mental and Physical Function

For the “maintenance of high mental and physical function” domain, the following five indicators were included based on the variables examined in previous studies [4,14,15,29]: activities of daily living (ADL), instrumental activities of daily living (IADL), six physical activities, cognitive function, and depressive symptoms. 

The criteria of satisfying each indicator were as follows. The subjects satisfied the criteria if they did not need help from others with respect to all seven and ten items of the ADL and IADL, respectively. For physical activities, the subjects satisfied the criteria if they could perform all of the following activities according to the senior fitness test developed by [30]; walking 400 m, climbing 10 stairs without stopping, bending over or crouching, reaching above head, sitting down and getting up five times from a chair without using two hands, and lifting or moving an 8 kg object. The cognitive function was measured by the Mini Mental State Examination for Dementia Screening (MMSE-DS) based on which the subjects satisfied the criteria if their cognitive functions were normal. Lastly, depressive symptoms were measured by the Short Geriatric Depression Scale-15 (SGDS-15) based on which the subjects satisfied the criteria if their score was less than eight [31]. 

It was classified as successful aging if the subjects satisfied at least three indicators out of the five indicators indicated by previous studies [15,32] in which the median value of indicators was set as the basis. 

(3) Continued Engagement with Life

The “continued engagement with life” domain was determined according to employment, participation in group activities, religious activities, volunteering activities, and lifelong learning based on the findings of previous studies [4,13,14,29]. It was classified as successful aging if the subjects satisfied at least three indicators out of the five indicators indicated by previous studies [16,32] in which the median value of indicators was set as the basis.

#### 2.3.2. Variables According to the Ecological System Model

(1) Individual System

The variables for the individual system included: age, sex, educational level, economic status, heavy drinking, smoking, exercise, subjective health status, and health screening. Age groups were divided into young-old adults (aged 65–74 years) and old-old adults (75 years or over). The educational level was reclassified into: lack of school, elementary school graduate, and middle school graduate or higher. The economic status was divided into five quantiles based on the income of all respondents of the 2017 NSLCKE: first quantile (7.54 million won or less), second quantile (7.55–12.08 million won), third quantile (12.09–19.46 million won), fourth quantile (19.47–34.26 million won), and fifth quantile (34.27 million won or more) [28]. The subjects were considered to be heavy drinkers if they drank seven glasses or more per week based on the threshold for moderate drinking; seven glasses per week was suggested in the 2017 NSLCKE. Subjective health status was reclassified; “very good” and “good” were re-coded as “good,” “fair” was coded “fair”, and “very poor” and “poor” were re-coded as “poor”. 

(2) Family System

The variables for the family system included living arrangement, satisfaction with spouse, satisfaction with children, frequency of contacting family, siblings, and relatives, and the number of close relatives including siblings. Living arrangements were classified into older adults living alone, older adults living with a spouse, and older adults living with children. The satisfaction with spouse and satisfaction with children were reclassified. “Very satisfied” and “satisfied” were re-coded as “satisfaction,” “average” was re-coded as “usual,” and “dissatisfied” and “very dissatisfied” were re-coded as “dissatisfaction.” The frequency of contacting family, siblings, and relatives was classified into less than once a month, between one and four times, and five times or more. 

(3) Community System

The variables for the community system included the frequency of contacting neighbors and friends, number of close neighbors and friends, and accessibility of neighborhood facilities. The frequency of contacting neighbors and friends was classified into less than once a month, between one and four times, and five times or more. The accessibility of neighborhood facilities was measured based on the time needed to arrive at six facilities (market/supermarket, hospital/clinic/health center, public office, senior welfare service center, other welfare service center, and bus stop/subway station), in which less than 5 min by walking was given 4 points, 5 to 10 min by walking was given 3 points, 10 to 30 min by walking was given 2 points, and more than 30 min by walking was given 1 point. The total score ranged from 6 to 24 points where a higher score indicated better access to facilities.

### 2.4. Data Analysis

The data were analyzed using the IBM SPSS statistical software, version 22 (IBM, Armonk, NY, USA). The variables for individual, family, and community systems were analyzed by descriptive statistics. The differences in successful aging by the variables were identified using the X^2^ test or t-test. Multivariate logistical regression analyses were conducted to identify the correlation factors of successful aging [33]. All *p* values <0.05 were considered to indicate statistical significance. 

## 3. Results 

The results of analyzing the differences in successful aging, the rate of successful aging, and the factors associated with successful aging according to the ecological system model among 10,074 subjects were as follows:

### 3.1. Differences in Successful Aging According to the Individual, Family, and Community System-Related Variables

The differences in successful aging according to the individual, family, and community system-related variables are shown in Table 2. All measured variables for individual, family, and community systems had a statistically significant difference in successful aging. 

### 3.2. Successful Aging in the Participants

Table 3 shows the rate of satisfaction for each domain and successful aging. 13.3% of subjects who were considered to have achieved successful aging met all three domains.

### 3.3. Factors Associated with Successful Aging

A multivariate logistical regression analysis was performed to identify the factors associated with successful aging. All variables confirmed to be significant in a univariate analysis were inputted for the analysis. The results are shown in Table 4. 

The factors associated with successful aging were: age, sex, educational level, economic status, heavy drinking, subjective health status, and health screening in the individual system; living arrangement, satisfaction with spouse, and frequency of contacting family, siblings, and relatives in the family system; and the frequency of contacting neighbors and friends, number of close neighbors and friends, and accessibility of neighborhood facilities in the community system.

The Hosmer–Lemeshow goodness of fit test had a *p*-value of 0.05 or greater, thus proving the goodness of fit of the regression model [33]. The Nagelkerke R^2^ value was 0.261, which indicated that this regression equation had an explanatory power of 26.1%. 

## 4. Discussion

This study was conducted to identify the factors associated with successful aging in older adults in Korea from a multidimensional perspective based on the ecological system model. The significance of this study is in that it confirmed that individual characteristics and the environmental systems surrounding older adults should be considered for successful aging. 

The study results showed that 13.3% of the subjects achieved successful aging. These results were similar to the results of a previous study on successful aging conducted among older adults [13], which reported 13.2% of subjects achieved successful aging, while it was higher than the 11.6% reported by a study conducted among older adults who used senior leisure and welfare facilities [14] and the 11.9% in a study conducted among older adults in the United States [34]. The results of this study were also lower than the 18.8% [29], 19.56% [15], and 23.1% [6] reported by previous studies conducted among older adults. The rate of successful aging varied by research mostly because the criteria for determining successful aging were different from each other. Therefore, an agreed concept of successful aging should be deduced in order to accurately reflect the concept.

The rate the domains of successful aging were satisfied was 44.6% for absence of disease and disease-related disability, 87.8% for maintenance of high mental and physical function, and 24.4% for continued engagement with life. The 44.6% for absence of disease and disease-related disability in this study was lower than the 71.30–84.31% which was reported in previous studies [14,15]. It can be inferred that the studies had different criteria in the evaluation of this domain. In this study, the subjects were examined if they had chronic diseases and disabilities and satisfied these criteria if they did not have both chronic diseases and disabilities. However, in a study by Han and Yoon [15], only one criterion of chronic diseases was applied, whereas, in a study by Lee and Song [14], the subjects satisfied the criteria if they had a normal BMI, did not smoke, and exercised even if they had chronic diseases. Therefore, the rate of the subjects satisfying the domain of absence of disease and disease-related disability was lower in this study. 

On the other hand, the rate of the subjects satisfying the domain of continued engagement with life was 24.4%, which was the lowest among the three domains. This result corresponds to the findings of previous studies [14,15], which also reported similar trends. The achievement rate of the “continued engagement with life” domain could be improved by encouraging the subjects to participate in volunteering or lifelong learning. If more volunteering opportunities are available and lifelong learning chances are provided to older adults who experience loss of role after retirement, these can help form a social support network that heightens social integration [35,36] and reduces aging anxiety [37]. Accordingly, the older adults can gain social health, which is part of total wellness, while improving the possibility of achieving successful aging.

The factors associated with successful aging according to the ecological system model included age, sex, educational level, economic status, heavy drinking, subjective health status, and health screening in the individual system. 

The male subjects had a 1.19 times higher chance of achieving successful aging, which was similar to the findings of previous studies [14,15]. Other studies [2,20] reported that gender was not a significant factor in successful aging, this was not consistent with our results. 

The possibility of achieving successful aging decreased as age increased, which was also observed in previous studies [13,20,32]. However, as aging will continue to progress rapidly, the older adult population will grow as well, thus requiring relevant measures. Measures to promote social participation while maintaining physical and cognitive functions in older adults should be explored in order to help them achieve successful aging despite growing older. In particular, health risk factors should be properly managed in order to maintain good physical health [8,32]. Based on the results of this study, which reported that the subjects who do not drink heavily had a 1.32 time higher chance of achieving successful aging, it can be inferred that drinking habits in older adults should be consistently managed. Temperance programs should be actively offered at community facilities, such as a senior citizen center or social welfare center, where many older adults visit. The older adult population should also be encouraged to participate in the temperance programs provided by the public health center. 

Moreover, a higher educational level and better economic status increased the possibility of achieving successful aging. This finding corresponds to the results of previous studies [20,38], where educational level and income level were the influential factors for successful aging. Educational level is often reported as a crucial factor for predicting the quality of life in old age [39]. More specifically, the education level of the older adults is one of the characteristics of a social class along with income. Hence, older adults with a higher educational level also have more active participation in economic activities and social networks in their later years, which positively affects life satisfaction [39]. Therefore, measures to improve successful aging in older adults with a low educational level and poor economic status should be derived. Establishing a lifelong learning system specially for older adults may supplement the low level of education. In terms of economics, quality employment opportunities for older adults should be actively discovered, while arranging measures for promoting economic stability of low-income households. 

Meanwhile, the subjects with a better subjective health status had a higher possibility of achieving successful aging, which was similar to the findings of previous studies [20,29,40,41]. Hence, the number of older adults who experience successful aging will increase if their subjective health can become more positive. Chronic disease and exercise are important factors affecting the subjective health status of older adults [40]. Individuals tend to perceive their health to be poor if they have chronic diseases [42]; however, their subjective health status can improve if their chronic diseases are managed well [41,43]. Hence, psychological and physical nursing interventions should be provided to help older adults perceive their health status more positively and to improve the ability of older adults to cope with and manage their chronic diseases. Furthermore, an exercise program that encourages consistent participation from older adults should be developed by considering their physical characteristics as they tend to perceive their health status to be better if they are actively engaged in exercises [40]. Consequently, their subjective health status can improve through such measures. 

The subjects who received a health screening had a 1.58 times higher chance of achieving successful aging. Due to the lack of previous studies examining the correlation between a health screening and successful aging, a direct comparison could not be made. However, receiving a health screening can be considered as the subjects’ willingness to manage their health [2]. It can be interpreted that older adults are taking initiative in their life and their strong will to improve their health may have affected the possibility of achieving successful aging. Accordingly, institutional measures or policies should be implemented to increase the rate of health screenings performed for older adults. The importance of a health screening should be promoted, and older adults should be informed of the nearby location of screening centers in the community where examinations are performed for their convenience. Such measures will improve the possibility of achieving successful aging, while at the same time, reducing the medical cost due to prevention and early detection of diseases [44]. 

The variables related to successful aging in the family system were living arrangement, satisfaction with spouse, and frequency of contacting family, siblings, and relatives. The subjects who have a satisfactory relationship with their spouse and those who live alone have a higher chance of achieving successful aging than those who live with their children. In other words, the relationship with a spouse, rather than children, has a greater impact in the family system. The result reflects the change in the lifestyle of older adults. Specifically, older adults prefer an independent relationship with children rather than depending on them in their later years. Hence, it is necessary to help older adults manage their own health and perceive themselves as healthy individuals, instead of depending on their children, for successful aging. Since a supportive relationship with a spouse has a positive influence on successful aging, when interventions are provided for older adults, it is advised that they should be encouraged to participate with their spouse or the programs should focus on improving the marital relationship. 

The variables related to successful aging in the community system were the frequency of contacting neighbors and friends, number of close neighbors and friends, and accessibility of neighborhood facilities. As reported in a previous study [34,45], the subjects who contact their neighbors and friends more frequently and who have a greater number of close neighbors and friends have a higher chance of achieving successful aging. Such social support is a positive resource that an individual can obtain from interpersonal relationships, thus maintaining a social support system in old age is particularly important for older adults who often experience a sense of loss [36]. Therefore, measures for strengthening social support to bring positive influence in the life of older adults and for enhancing the experience of successful aging are needed. Based on the findings of previous studies [46], which reported that boosting self-confidence was more effective for successful aging than providing only materialistic support and information to older adults by considering them as mere recipients of social support, relevant measures should be established to help older adults be conscious of themselves as valuable and independent beings. One of the effective strategies can be providing support which directly expresses genuine understanding of and interest in older adults and accepts them while giving positive judgments. Strengthening a social support system centered around families is limited since nuclear families are becoming a social norm; thus, various types of emotional support involving friends, communities, organizations, and experts, in addition to a system for heightening self-esteem, should be established and reinforced. 

Meanwhile, higher accessibility to neighborhood facilities had a 0.95 time lower chance of achieving successful aging. This result is in line with a previous study, which reported that no difference was observed according to the accessibility in the community environment [47], while other studies [41,48] have reported that the possibility of achieving successful aging is higher when facilities are located conveniently in areas where older adults inhabit, thus showing discrepancies in the results. However, the community environment is a factor that directly affects successful aging in older adults [48], hence encouraging older adults to participate in social activities if the accessibility to neighborhood facilities is easy and can be expected to have a positive effect on successful aging. Therefore, further studies are needed to examine the accessibility to neighborhood facilities. 

This study was limited in that it used secondary data that did not include psychological and subjective indicators, such as psychological well-being, efficacy, and self-control, which may have affected successful aging [2,29], when selecting the variables. Therefore, studies on successful aging in older adults should be conducted in the future to include those factors. 

## 5. Conclusions

This study analyzed the factors for successful aging in older adults in Korea based on the ecological system model. The factors associated with successful aging were: age, sex, educational level, economic status, heavy drinking, subjective health status, and health screening in the individual system; living arrangement, satisfaction with spouse, and the frequency of contacting family, siblings, and relatives in the family system; and the frequency of contacting neighbors and friends, number of close neighbors and friends, and accessibility of neighborhood facilities in the community system. It was confirmed that individual characteristics, as well as environmental systems surrounding the older adults, should be considered comprehensively for the successful aging of older adults. 

In the future, studies should be conducted to examine the influential factors for successful aging based on subjective indicators that have not been investigated in-depth in this study. 

In addition, based on the results of this study, it is necessary to develop educational or nursing intervention programs for successful aging that consider not only individual aspects but also the community environment. Furthermore, age-friendly environments should be developed and improved.

## Figures and Tables

**Table 1:** Operational Definition of Successful Aging based on Rowe and Kahn’s Model.

Domain	Indicators	Met Criteria
Absence of disease and disease-related disability	Number of chronic diseases ≤2	Satisfied two indicators in this domain
	No disability	
Maintenance of high mental and physical function	Do all ADL	Satisfied at least three indicators in this domain
	Do all IADL	
	Do all 6 physical activities	
	MMSE-DS score: normal	
	SGDS-15 score: under 8 points	
Continued engagement with life	Employment	Satisfied at least three indicators in this domain
	Participation in group activities	
	Participation in religious activities	
	Participation in volunteering activities	
	Participation in lifelong learning	

ADL = Activities of Daily Living; IADL = Instrumental Activities of Daily Living; MMSE-DS = Mini Mental State Examination for Dementia Screening; SGDS-15 = Short Geriatric Depression Scale-15.

**Table 2:** The Differences in Successful Aging According to the Individual, Family, and Community System-related Variables. *N* = 10,074.

Level	Variables	Categories	Successful Aging (n = 1343)	Usual Aging (n = 8731)	X^2^ (*p*)
			n (%) or Mean ± SD	n (%) or Mean ± SD	
Individual	Age (year)	65–74	1086 (18.6)	4764 (81.4)	330.64 ***
		≥75	257 (6.1)	3967 (93.9)	
	Sex	Male	761 (17.8)	3524 (82.2)	127.05 ***
		Female	581 (10.0)	5206 (90.0)	
	Educational level	No education	105 (4.4)	2288 (95.6)	372.09 ***
		Elementary school	372 (10.8)	3080 (89.2)	
		≥Middle school	866 (20.5)	3363 (79.5)	
	Economic Status	1st quintile	39 (4.1)	906 (95.9)	271.99 ***
		2nd quintile	129 (6.6)	1822 (93.4)	
		3rd quintile	275 (11.9)	2032 (88.1)	
		4th quintile	410 (16.8)	2027 (83.2)	
		5th quintile	489 (20.1)	1943 (79.9)	
	Heavy drinking	Yes	842 (11.1)	6775 (88.9)	140.35 ***
		No	501 (20.4)	1955 (79.6)	
	Smoking	Yes	173 (16.8)	856 (83.2)	12.02 **
		No	1170 (12.9)	7875 (87.1)	
	Exercise	Yes	1028 (15.0)	5826 (85.0)	51.59 ***
		No	315 (9.8)	2905 (90.2)	
	Subjective health status	Good	955 (25.6)	2770 (74.4)	850.19 ***
		Fair	257 (10.9)	2092 (89.1)	
		Poor	131 (3.3)	3870 (96.7)	
	Health screening	Yes	1209 (14.5)	7146 (85.5)	54.87 ***
		No	134 (7.8)	1584 (92.2)	
Family	Living arrangement	Living alone	189 (7.8)	2227 (92.2)	104.47 ***
		Living with spouse	805 (16.4)	4103 (83.6)	
		Living with children	349 (12.7)	2401 (87.3)	
	Satisfaction with spouse + (n = 6404)	Satisfaction	863 (18.8)	3734 (81.2)	49.63 ***
		Usual	150 (10.8)	1237 (89.2)	
		Dissatisfaction	62 (14.8)	358 (85.2)	
	Satisfaction with children+ (n = 9839)	Satisfaction	1134 (15.0)	6414 (85.0)	64.46 ***
		Usual	149 (9.1)	1485 (90.9)	
		Dissatisfaction	47 (7.2)	610 (92.8)	
	Contact with family/siblings/relatives (time/month)	<1	527 (10.2)	4639 (89.8)	94.78 ***
		1–4	474 (15.8)	2531 (84.2)	
		≥5	342 (18.0)	1561 (82.0)	
	Number of close relatives		1.15 ± 1.48	0.79 ± 1.18	−8.70 ***
Community	Contact with friends/neighbors (time/month)	<1	13 (2.0)	624(98.0)	105.34 ***
		1–4	36 (6.4)	525 (93.6)	
		≥5	1293 (14.6)	7581 (85.4)	
	Number of close friends/neighbors		2.14 ± 2.23	1.33 ± 1.85	−12.64 ***
	Accessibility to neighborhood facilities		12.60±3.05	12.86 ± 2.88	2.93 **

** *p* < 0.01, *** *p* < 0.001. + missing data.

**Table 3:** The Proportion of Successful Aging, *N* = 10,074.

Domains	n (%)
Absence of diseases and disease-related disability	4497 (44.6)
Maintenance of high mental and physical function	8844 (87.8)
Continued engagement with life	2456 (24.4)
Successful aging	1343 (13.3)

**Table 4:** Logistic Regression for Successful Aging related Variables, *N* = 10,074.

Level	Variables	Categories	Successful Aging
			OR (95% CI)
Individual	Age (year)	65–74	2.18 (1.86–2.55) ***
		≥75	1
	Sex	Male	1.19 (1.02–1.38) *
		Female	1
	Educational level	No education	1
		Elementary school	1.44 (1.13–1.83) **
		≥Middle school	2.13 (1.68–2.70) ***
	Economic Status	1st quintile	1
		2nd quintile	1.29 (0.87–1.90)
		3rd quintile	1.79 (1.23–2.61) **
		4th quintile	2.33 (1.59–3.40) ***
		5th quintile	2.55 (1.73–3.76) ***
	Heavy drinking	Yes	1
		No	1.32 (1.15–1.52) ***
	Subjective health status	Good	6.33 (5.21–7.70) ***
		Fair	2.73 (2.18–3.41) ***
		Poor	1
	Health screening	Yes	1.58 (1.29–1.93) ***
		No	1
Family	Living arrangement	Living alone	1.54 (1.14–2.08) *
		Living with spouse	1.11 (0.93-1.33)
		Living with children	1
	Satisfaction with spouse	Satisfaction	1.81 (1.22–2.68) **
		Usual	1.08 (0.79–1.49)
		Dissatisfaction	1
	Contact with family/siblings/relatives (time/month)	<1	1
		1–4	1.14 (0.99–1.32)
		≥5	1.32 (1.12–1.56) ***
Community	Contact with friends/neighbors (time/month)	<1	1
		1–4	2.33 (1.20–4.52) *
		≥5	4.10 (2.33–7.22) ***
	Number of close friends/neighbors		1.06 (1.03–1.09) ***
	Accessibility to neighborhood facilities		0.95 (0.93–0.97) ***
(Constant)		0.01 ***	
Correct prediction (%)		86.9	
Hosmer–Lemeshow test		x^2^ = 6.99, df = 8, *p* = 0.538	
Nagelkerke R^2^		0.261	

* *p* < 0.05, ** *p* < 0.01, *** *p* < 0.001.
